# Short-term and long-term reversion rates to normal cognition and their contributing factors among individuals with mild cognitive impairment in a Japanese community: the Hisayama study

**DOI:** 10.1186/s12877-025-06750-7

**Published:** 2025-12-29

**Authors:** Kaishi Takabatake, Tomoyuki Ohara, Toshifumi Minohara, Taro Nakazawa, Emi Oishi, Yoshihiko Furuta, Satoko Sakata, Mao Shibata, Tomohiro Nakao, Toshiharu Ninomiya

**Affiliations:** 1 Department of Neuropsychiatry, Graduate School of Medical Sciences, Kyushu University, Fukuoka, Japan; 2 Department of Epidemiology and Public Health, Graduate School of Medical Sciences, Kyushu University, Fukuoka, Japan; 3 Center for Cohort Studies, Graduate School of Medical Sciences, Kyushu University, Fukuoka, Japan; 4 Department of Medicine and Clinical Science, Graduate School of Medical Sciences, Kyushu University, Fukuoka, Japan; 5 Department of Psychosomatic Medicine, Graduate School of Medical Sciences, Kyushu University, Fukuoka, Japan

**Keywords:** Mild cognitive impairment, Reversion, Normal cognition, Dementia, Risk factors

## Abstract

**Background:**

Individuals with mild cognitive impairment (MCI) have a high risk of developing dementia, but some remain cognitively stable or even revert to normal cognition (NC), with reported reversion rates varying widely and inconsistent findings regarding contributing factors. The purposes of this study were to investigate the reversion rate from MCI to NC in a community-dwelling older Japanese population and to identify factors associated with reversion.

**Methods:**

Three community-based surveys of cognitive function were conducted in the town of Hisayama among residents aged 65 years or older in 2012–2013, 2017–2018, and 2022–2023 (participation rate > 92% for each time range). The 5-year reversion rate from MCI to NC was examined using a pooled cohort, combining two cohorts from the 2012–2013 to the 2017–2018 survey and from the 2017–2018 to the 2022–2023 survey. A logistic regression analysis with generalized estimating equations was used to identify factors associated with reversion to NC based on the pooled cohort data. To evaluate the 10-year reversion rate of MCI, moreover, 171 participants diagnosed with MCI in the 2012–2013 survey were followed for 10 years.

**Results:**

The 5-year reversion rate from MCI to NC in the pooled cohort was 31.3% (95% confidence interval, 26.9%–36.2%). Younger age, lower systolic blood pressure, absence of diabetes mellitus, absence of instrumental activities of daily living impairment, higher handgrip strength, higher Mini-Mental State Examination (MMSE) score, larger total brain volume, and smaller cerebral white matter lesion volume were significantly associated with reversion to NC. Meanwhile, the 10-year reversion rate from MCI to NC was 18.1% (13.1%–24.6%). Of individuals who reverted from MCI to NC in the 2017–2018 survey, 48.1% (35.0%–61.5%) maintained NC five years later, 32.7% (21.4%–46.4%) returned to MCI, and 5.8% (1.9%–16.4%) progressed to dementia.

**Conclusions:**

In a Japanese community-dwelling older population with MCI, approximately 30% and 18% reverted to NC for 5 years and 10 years, respectively. The prevention and proper management of lifestyle-related diseases and promotion of healthy lifestyle behaviors may play a crucial role in facilitating reversion to NC in older individuals with MCI.

**Supplementary Information:**

The online version contains supplementary material available at 10.1186/s12877-025-06750-7.

## Background

The global prevalence of mild cognitive impairment (MCI) and dementia in older individuals is expected to rise steadily, posing significant socioeconomic, medical, and public health concerns [1]. MCI is considered to be an early stage of dementia because population-based epidemiological studies have shown that the risk of developing dementia is significantly higher in participants with MCI than those with normal cognition (NC) [2, 3]. However, it has also been reported that individuals with MCI do not necessarily progress to dementia, and their cognitive state may remain stable or even return to NC over time [4]. Several epidemiological longitudinal studies have examined the reversion rate from MCI to NC, but the rates varied across the studies, ranging from 8.5% to 44.0% [5–12]. In addition, most of these previous studies followed MCI participants for relatively short periods (less than 5 years); long-term longitudinal studies are limited, with only one retrospective study following participants for more than 10 years [12]. Moreover, despite the inconsistent results in previous studies [5–11, 13–17], identifying the factors that contribute to reversion to NC in individuals with MCI could enhance our understanding of cognitive reserve and help to reduce the societal burden of cognitive impairment.

The Hisayama Study is a community-based prospective longitudinal study of cardiovascular diseases that has been ongoing in the town of Hisayama, Japan [18, 19]. Since 1985, repeated community-based surveys on dementia have also been conducted, with the evaluation of MCI additionally included since 2012 [20, 21]. The purposes of this study were to investigate the 5-year reversion rate from MCI to NC in a community-dwelling older Japanese population, as well as to identify the factors associated with reversion to NC. Finally, using 10-year follow-up data, the long-term prognosis was examined, including the cognitive trajectory after reversion to NC.

## Methods

### Study population

In the Hisayama Study, all residents aged 65 years or older in the town of Hisayama, Fukuoka, Japan, were invited to participate in the comprehensive survey of cognitive function. Individuals with psychiatric disorders, intellectual disabilities, or consciousness disturbances were excluded. These full community-based surveys on dementia were repeatedly conducted in 1985, 1992, 1998, 2005, 2012–2013, 2017–2018, and 2022–2023 with the same uniform screening methods and diagnostic criteria [20, 21]. Beginning in 2012–2013, an evaluation of MCI was also included. In the present study, the data from the surveys of MCI and dementia conducted in 2012–2013, 2017–2018, and 2022–2023 were used. In the 2012–2013 survey, a total of 1,906 individuals (780 men and 1,126 women) among 2,036 residents aged 65 or older participated (participation rate 94.6%) and 187 of them (prevalence 9.8%) were diagnosed with MCI. In a similar manner, 2,202 (932 men and 1,270 women) of 2,340 residents (participation rate 94.1%) participated in the 2017–2018 survey, and 2,302 (979 men and 1,323 women) of 2,422 residents (participation rate 95.0%) participated in the 2022–2023 survey; the numbers of participants diagnosed with MCI in these surveys were 211 (9.6%) and 269 (11.7%), respectively.

**Fig. 1:**
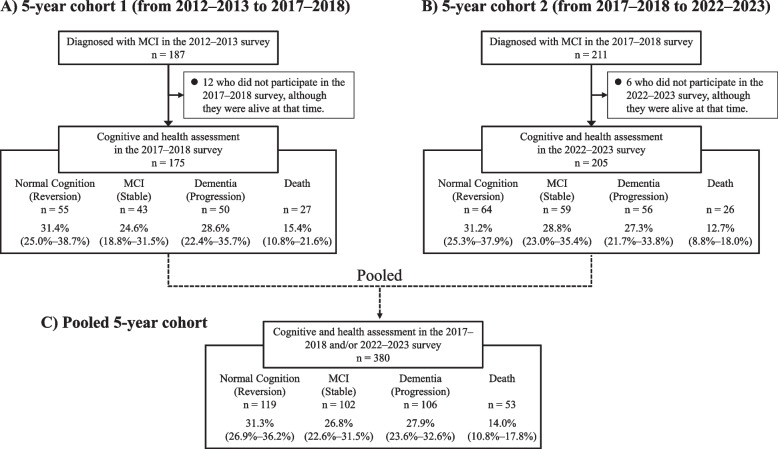
Five-year change rates in cognitive status and mortality rate among participants with MCI. Abbreviations: MCI, mild cognitive impairment.The values in parentheses indicate the 95% confidence intervals for each change rate

For the analysis of 5-year reversion rates from MCI to NC and the contributing factors associated with the reversion, two 5-year cohorts were established. For the first cohort, among 187 individuals with MCI in the 2012–2013 survey, 175 individuals who underwent cognitive and health assessments, including information on survival status, in the 2017–2018 survey were included (hereafter referred to as the 5-year cohort 1), after excluding 12 individuals who did not participate in the 2017–2018 survey, although they were alive at that time (Fig. 1A). Similarly, among 211 individuals with MCI in the 2017–2018 survey, 205 individuals who underwent cognitive and health assessments in the 2022–2023 survey were included in the second cohort (hereafter referred to as the 5-year cohort 2) (Fig. 1B). In addition, a total of 380 individuals with MCI were included by pooling the data from these two 5-year cohorts (Fig. 1C).

**Fig. 2:**
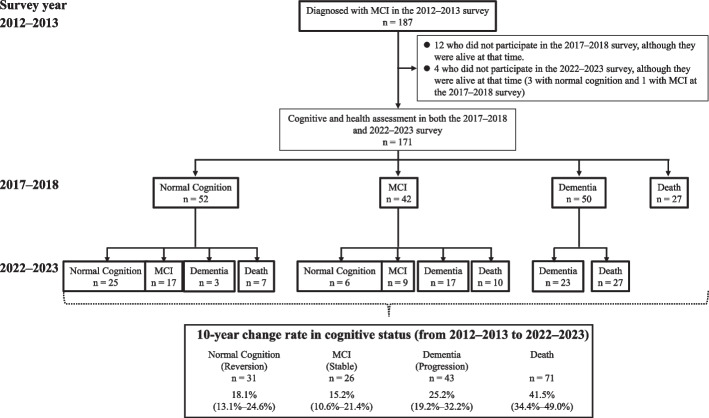
Ten-year change rates in cognitive status and mortality rate among participants with MCI. Abbreviations: MCI, mild cognitive impairment. The values in parentheses indicate the 95% confidence intervals for each change rate

For the analysis of 10-year reversion rates from MCI to NC, a cohort followed longitudinally from the 2012–2013 survey to the 2022–2023 survey was also established (hereafter referred to as the 10-year cohort). This cohort included 171 individuals who were diagnosed with MCI at the 2012–2013 survey and underwent cognitive and health assessment in both the 2017–2018 and 2022–2023 surveys were included in the cohort, for which 12 individuals who did not participate in the 2017–2018 survey and 4 individuals (3 with NC and 1 with MCI at the 2017–2018 survey) who did not participate in the 2022–2023 survey were excluded from 187 individuals with MCI at the 2012–2013 survey (Fig. 2).

### Diagnosis of MCI and dementia

In each survey, a two-stage prevalence assessment of dementia and MCI was conducted. In the first screening, the Mini-Mental State Examination (MMSE) was performed [22, 23]. A licence to use the MMSE was obtained from Nihon Bunka Kagakusha with permission from Psychological Assessment Resources, Inc. When the test scores were lower than the cutoff points of 26 or subjective/objective cognitive impairment was suspected, a second-stage assessment was conducted by expert psychiatrists on the study team using a comprehensive battery of investigations, including the Wechsler Memory Scale of logical memory [24]. A cutoff score of 26 on the MMSE was adopted in accordance with previous studies indicating that this threshold provides reasonable sensitivity and specificity for detecting mild cognitive impairment in community-dwelling older adults [25]. The diagnosis of dementia and MCI were made using the clinical criteria of the Diagnostic and Statistical Manual of Mental Disorders, Third Edition, Revised [26] and the clinical criteria reported by Petersen et al. in 2001, [27] respectively. We defined MCI as objective cognitive impairment based on the neuropsychological data, and no participants were diagnosed as having subjective cognitive impairment. Expert psychiatrists and stroke physicians on the study team adjudicated every case of dementia and MCI. For all participants who died between surveys, the information on death or any cognitive impairment was collected through the daily monitoring system of the Hisayama Study, regular health checkups, and postal and telephone survey, as previously reported [19]. When information indicating a diagnosis of MCI or dementia before death was obtained, the individual was classified into the corresponding cognitive impairment group at the subsequent survey to reflect their cognitive status before death.

### Definition of risk factors

Each participant completed a self-administered questionnaire that included information on their treatment history (including medications for hypertension, diabetes mellitus, and dyslipidemia), medical history, educational level, smoking habit, alcohol intake, and regular exercise; all of which had been developed in our study (Supplemental information). Blood pressure was measured three times with an automated sphygmomanometer after an at least 5-min rest in the sitting position. The mean of the three measurements was used in the analyses. Diabetes mellitus was defined as fasting plasma glucose ≥ 7.0 mmol/L, 2-h postload or casual glucose ≥ 11.1 mmol/L, or current treatment with oral antidiabetic agents or insulin. Serum total cholesterol levels were measured enzymatically. Height and weight were measured in light clothing without shoes, and body mass index (BMI) was calculated. BMI < 18.5 kg/m^2^ was defined as leanness. ADL impairment was assessed using the Barthel Index, with a score ≤ 95 points indicating impairment, as reported in previous studies [28, 29]. IADL impairment was evaluated using the IADL scale from the Institute of Gerontology [30], which has been validated for use in Japan [31], and was defined as the inability to perform at least 1 of the 13 items on the scale, following the definition used in previous studies [32–34]. Educational level was categorized as either ≤ 9 years or ≥ 10 years of formal education. Smoking habit and alcohol intake were classified as either present or not. Regular exercise was defined as engaging in sports or other physical exercise including recreational walking at least three times a week during leisure time. Handgrip strength was measured using a digital strength dynamometer (T.K.K.5401; Takei Scientific Instruments, Niigata, Japan) under the guidance of trained personnel or a nurse. Measurements were taken twice for each hand in an alternating manner, and the highest value among the four trials was used for analysis. Depressive symptoms were defined as a score of ≥ 6 on the Geriatric Depression Scale (GDS) [35]. To determine the APOE polymorphism, two single nucleotide polymorphisms (rs429358 and rs7412) were genotyped using the multiplex polymerase chain reaction–based Invader assay [36] or the multiplex polymerase chain reaction-based targeted sequencing method [37] as previously reported.

### MRI examination in the 2012–2013 and 2017–2018 surveys

As part of the 2012–2013 and 2017–2018 surveys, brain magnetic resonance imaging (MRI) was also performed. Among total participants with MCI in the 2012–2013 survey, 145 (82.9%) underwent MRI, while 172 (83.9%) participants with MCI underwent MRI in the 2017–2018 survey. Using a 1.5-Tesla MRI scanner (Intera Pulsar; Philips Medical Systems, Best, the Netherlands) with a multichannel head coil, we examined 3-dimensional T1-weighted images, conventional T1- and T2-weighted images, fluid-attenuated inversion recovery (FLAIR) images, T2*-weighted images, and magnetic resonance angiographic images of the brain. White matter lesions on T1‐weighted and FLAIR images were segmented using the Lesion Segmentation Toolbox for SPM12 with 0.15 as the threshold of signal intensity (κ value), as described previously [38]. The 3-dimensional T1-weighted images were converted to Neuroimaging Informatics Technology Initiative format and then segmented into three components (grey matter, white matter and cerebrospinal fluid) by using VBM8 Toolbox version 435 (University of Jena, Germany) in SPM8 (University College London, UK) running on MATLAB (The Mathworks, Natick, Massachusetts, USA). The International Consortium for Brain Mapping template for East Asian brains was used for anatomical settings. The total brain volume (TBV) was calculated as the sum of gray and white matter volumes, and the intracranial volume (ICV) as the sum of TBV and cerebrospinal fluid volumes. The TBV/ICV (%) was calculated as the ratio of TBV to ICV and used as an index of total brain volume corrected for ICV, while the ratio of white matter lesion volume (WMLV) to ICV (WMLV/ICV %) was used as an index of white matter lesions corrected for ICV. The WMLV/ICV levels were natural log-transformed when used as a continuous variable because their distribution was skewed.

### Statistical analysis

The baseline characteristics of participants in the 2012–2013 and the 2017–2018 cohort were compared using a t-test for continuous variables and χ^2^test for categorical variables. A logistic regression model was used to calculate the mortality rate and change rates in the three cognitive statuses of NC, MCI, and dementia over the follow-up period, along with their 95% confidence intervals (CIs). To identify contributing factors associated with reversion from MCI to NC, a logistic regression analysis with generalized estimating equations was used, taking into account duplicate participants who were included in both cohorts [39]. In these analyses, the reference group was defined as participants who remained with MCI during follow-up, progressed to dementia, or died before the follow-up assessment. Under the assumptions of a total sample size of 380, an exposure prevalence of 30%, a cumulative incidence of reversion of 30% in the reference group, a two-sided α of 0.05, and an odds ratio of 2.0 to be detected, the statistical power of this analysis was approximately 0.85. In the age- and sex-adjusted analysis, a false discovery rate (FDR) correction using the Benjamini–Hochberg method was applied to account for multiple testing, and the corresponding FDR q-values were calculated [40]. We also performed multivariable-adjusted analyses including demographic, lifestyle, and health-related factors, using a backward elimination method with a retention criterion of p < 0.10. When applying the Fine–Gray competing risk model [41], the exact date of death was available, allowing the use of true time-to-event data. In contrast, the time variable for cognitive status was calculated as the difference between the dates of the two time points at which cognitive status was assessed, and tied event times were handled using the exact method in the relevant model. In these analyses, only complete cases were included after excluding participants with missing values. All statistical analyses were performed using SAS version 9.4 (SAS Institute Inc., Cary, NC, USA). Two-sided values of p < 0.05 and FDR-q < 0.10 were considered statistically significant in all analyses.

## Results

Figure 1 shows the 5-year change rates for each cognitive status and the mortality rate among participants with MCI in the 5-year cohorts 1 and 2. The baseline characteristics of participants with MCI were generally similar between the two cohorts, with a few exceptions: the frequency of leanness, the frequency of completing ≤ 9 years of education and the mean MMSE score differed significantly between the two cohorts (Table 1). In 5-year cohort 1, among the 175 participants with MCI, 55 participants (31.4% [95% CI, 25.0%–38.7%]) reverted to NC, 43 (24.6% [18.8%–31.5%]) continued to exhibit MCI, 50 (28.6% [22.4%–35.7%]) progressed to dementia, and 27 (15.4% [10.8%–21.6%]) died during the follow-up period (Fig. 1A). Similarly, in 5-year cohort 2, among the 205 participants with MCI, 64 (31.2% [25.3%–37.9%]) reverted to NC, 59 (28.8% [23.0%–35.4%]) continued to exhibit MCI, 56 (27.3% [21.7%–33.8%]) progressed to dementia, and 26 (12.7% [8.8%–18.0%]) died (Fig. 1B). There was no evidence of a significant difference in the rates of change in cognitive status or mortality between the two cohorts (all p > 0.90 for NC, MCI, dementia, and death). As similar change rates were observed between the two cohorts, the two 5-year cohorts were pooled to increase the sample size for the analysis. As a consequence, the 5-year reversion rate from MCI to NC was 31.3% (95% CI, 26.9%–36.2%), while 26.8% (22.6%–31.5%) continued to have MCI, 27.9% (23.6%–32.6%) progressed to dementia, and 14.0% (10.8%–17.8%) died during the follow-up period (Fig. 1C). We additionally performed a subgroup analysis of the impact of age (< 75 and ≥ 75 years) using the pooled 5-year cohort (Table 2). Among participants aged < 75 years, the 5-year reversion rate from MCI to NC was 51.2% (42.4%–59.9%), while 26.8% (19.8%–35.3%) continued to have MCI, 14.6% (9.4%–22.0%) progressed to dementia, and 7.3% (3.9%–13.5%) died during the follow-up period. Among those aged ≥ 75 years, the 5-year reversion rate from MCI to NC was 21.8% (17.2%–27.3%), while 26.9% (21.8%–32.6%) continued to have MCI, 34.2% (28.7%–40.3%) progressed to dementia, and 17.1% (13.0%–22.0%) died during the follow-up period.

**Table 1:** Clinical characteristics of participants with MCI in the 2012–2013 and 2017–2018 surveys

Variables	Survey year		*p*-value	N (%) of missing data
	2012–2013 (*n* = 175)	2017–2018 (*n* = 205)		
MMSE score, mean (SD)	25.0 (2.7)	24.2 (2.6)	0.005	9 (2.4)
***Demographic, lifestyle, and health-related factors***				
Age, mean (SD), years	77.3 (6.7)	78.1(7.2)	0.28	0 (0.0)
Female, %	51.4	53.2	0.73	0 (0.0)
Low education level (≤ 9 years), %	50.3	40.1	0.049	8 (2.1)
Systolic blood pressure, mean (SD), mmHg	134.7 (20.3)	135.1 (22.1)	0.84	20 (5.3)
Use of antihypertensive medication, %	61.2	56.1	0.33	19(5.0)
Diabetes mellitus, %	28.5	25.0	0.46	19 (5.0)
Serum total cholesterol, mean (SD), mg/dL	191.5 (34.6)	197.7 (39.3)	0.11	20 (5.3)
Use of lipid-modifying medication, %	33.9	34.2	0.96	19(5.0)
History of cardiovascular disease, %	15.4	19.0	0.36	0 (0.0)
BMI, mean (SD), kg/m^2^	23.1 (3.3)	23.0 (3.9)	0.65	19 (5.0)
BMI < 18.5 kg/m^2^, %	4.2	11.7	0.01	19 (5.0)
ADL disability, %	13.1	13.8	0.85	2 (0.5)
IADL disability, %	49.7	58.1	0.10	0 (0.0)
Ever smoking habit (Current or Former), %	43.6	41.0	0.62	20 (5.3)
Ever alcohol intake (Current or Former), %	58.2	62.6	0.40	20 (5.3)
Regular exercise, %	42.4	35.8	0.20	22 (5.8)
Handgrip strength, mean (SD), kg	26.6 (8.3)	26.1 (8.6)	0.63	40 (10.5)
Depressive symptoms, %	24.4	26.2	0.70	13 (3.4)
APOE-ε4 carrier, %	19.8	17.3	0.55	11 (2.9)
***Brain imaging factors***				
TBV/ICV, mean (SD), %	66.8 (3.8)	66.2 (4.1)	0.13	63 (16.6)
WMLV/ICV, geometric mean (geometric SD), %	0.29 (1.24)	0.27 (1.24)	0.43	63 (16.6)

*Abbreviations*: *MCI* mild cognitive impairment, *SD* standard deviation, *MMSE* Mini-Mental State Examination, *BMI* body mass index, *ADL* activities of daily living, *IADL* instrumental activities of daily living, *APOE* apoprotein E, *TBV* total brain volume, *ICV* intracranial volume, *WMLV* white matter lesion volume

**Table 2:** Subgroup analysis of the 5-year change rates of each cognitive status and mortality by age

Cognitive and health status	No. of events	5-year change rate (95% CI), %
Age < 75 years (*n* = 123)		
Normal cognition	63	51.2 (42.4–59.9)
Mild cognitive impairment	33	26.8 (19.8–35.3)
Dementia	18	14.6 (9.4–22.0)
Death	9	7.3 (3.9–13.5)
Age ≥ 75 years (*n* = 257)		
Normal cognition	56	21.8 (17.2–27.3)
Mild cognitive impairment	69	26.9 (21.8–32.6)
Dementia	88	34.2 (28.7–40.3)
Death	44	17.1 (13.0–22.0)

*Abbreviations*: *CI* confidence interval

Next, we investigated the factors associated with reversion from MCI to NC using the pooled 5-year follow-up data of participants with MCI. The unadjusted cumulative incidence rates of reversion from MCI to NC according to the presence or absence of demographic, lifestyle, and health-related factors were shown in Table S1. As shown in Table 3, younger age, lower systolic blood pressure, absence of diabetes mellitus, absence of IADL impairment, higher handgrip strength, higher MMSE score, higher TBV/ICV levels, and lower WMLV/ICV levels were significantly associated with higher age- and sex-adjusted odds ratios of reversion from MCI to NC. FDR correction did not alter the findings substantially. No significant heterogeneity was detected in the magnitude of the association between each factor and reversion from MCI to NC (all p > 0.15 for heterogeneity). The sensitivity analysis after excluding deceased cases (Table S2) and using a competing risk model for dementia and death (Table S3) did not materially alter the results. In addition, we have performed the multivariable-adjusted analysis including demographic, lifestyle, and health-related factors-namely, age, sex, education level, systolic blood pressure, use of antihypertensive medication, diabetes mellitus, serum total cholesterol, use of lipid-modifying medication, history of cardiovascular disease, BMI < 18.5 kg/m^2^, ADL disability, IADL disability, smoking habit, alcohol intake, regular exercise, handgrip strength, depressive symptoms, and APOE-ε4 carrier as covariates (Table 4). After selecting the variable using a backward elimination method, younger age, female sex, lower systolic blood pressure, use of antihypertensive medication, no diabetes mellitus, no IADL disability, and higher handgrip strength were selected as factors related to reversion from MCI to NC.

**Table 3:** Age- and sex-adjusted odds ratios of reversion from MCI to NC for each risk factor

Variables	Units	Age- and sex-adjusted OR (95% Cl) of reversion	*p*-value	FDR q value
MMSE score	(per 1 point increase)	1.13 (1.01–1.26)	0.03	0.09
***Demographic, lifestyle, and health-related factors***				
Age	(per 1 year decrease)	1.12 (1.08–1.16) ^a)^	< 0.001	< 0.001
Sex	(Female vs. Male)	1.06 (0.66–1.68) ^b)^	0.82	0.86
Education level (≤ 9 years)	(≥ 10 years vs. ≤ 9 years)	1.04 (0.64–1.70)	0.88	0.88
Systolic blood pressure	(per 10 mmHg decrease)	1.14 (1.02–1.27)	0.02	0.08
Use of antihypertensive medication	(Yes vs. No)	1.47 (0.90–2.41)	0.13	0.27
Diabetes mellitus	(No vs. Yes)	1.92 (1.09–3.40)	0.02	0.07
Serum total cholesterol	(per 10 mg/dL decrease)	1.06 (0.99–1.13)	0.07	0.17
Use of lipid-modifying medication	(Yes vs. No)	1.25 (0.76–2.07)	0.38	0.53
History of cardiovascular disease	(No vs. Yes)	1.12 (0.59–2.14)	0.73	0.81
BMI < 18.5 kg/m^2^	(No vs. Yes)	1.55 (0.66–3.64)	0.32	0.47
ADL disability	(No vs. Yes)	1.49 (0.71–3.11)	0.29	0.47
IADL disability	(No vs. Yes)	1.74 (1.11–2.73)	0.02	0.08
Smoking habit	(Never vs. Ever)	1.13 (0.63–2.01)	0.69	0.80
Alcohol intake	(Never vs. Ever)	0.70 (0.41–1.20)	0.19	0.34
Regular exercise	(Yes vs. No)	1.15 (0.72–1.86)	0.55	0.68
Handgrip strength	(per 1 kg increase)	1.10 (1.04–1.16)	< 0.001	0.004
Depressive symptoms	(No vs. Yes)	1.47 (0.83–2.60)	0.19	0.37
APOE-ε4 carrier	(No vs. Yes)	0.78 (0.43–1.40)	0.40	0.52
***Brain imaging data***				
TBV/ICV	(per 1 unit increase)	1.20 (1.10–1.32)	< 0.001	< 0.001
WMLV/ICV	(per 1 unit decrease)	1.56 (1.07–2.26)	0.02	0.07

*Abbreviations*: *OR* odds ratio, *CI* confidence interval, *MCI* mild cognitive impairment, *MMSE* Mini-Mental State Examination, *NC* normal cognition, *BMI* body mass index, *ADL* activities of daily living, *IADL* instrumental activities of daily living, *APOE* apoprotein E, *TBV* total brain volume, *ICV* intracranial volume, *WMLV* white matter lesion volume

^a)^ This value is adjusted for sex

^b)^ This value is adjusted for age

**Table 4:** Multivariable-adjusted odds ratios of reversion from MCI to NC for each risk factor

Variables	Units	Multivariable-adjusted model (Full model)		Multivariable-adjusted model (using a backward elimination method)^a)^	
		OR (95%CI) of reversion	*p*-value	OR (95%CI) of reversion	*p*-value
Age	(per 1 year decrease)	1.08 (1.03–1.13)	0.003	1.10 (1.04–1.15)	< 0.001
Sex	(Female vs. Male)	4.14 (1.26–13.62)	0.02	3.42 (1.31–8.90)	0.01
Education level	(≥ 10 years vs. ≤ 9 years)	1.03 (0.58–1.84)	0.93		
Systolic blood pressure	(per 10 mmHg decrease)	1.13 (1.00–1.27)	0.048	1.11 (0.99–1.25)	0.07
Use of antihypertensive medication	(Yes vs. No)	1.75 (0.93–3.29)	0.08	2.07 (1.19–3.61)	0.01
Diabetes mellitus	(No vs. Yes)	2.59 (1.31–5.14)	0.01	2.39 (1.26–4.52)	0.01
Serum total cholesterol	(per 10 mg/dL decrease)	1.05 (0.97–1.12)	0.22		
Use of lipid-modifying medication	(Yes vs. No)	1.28 (0.71–2.30)	0.41		
History of cardiovascular disease	(No vs. Yes)	1.55 (0.68–3.51)	0.29		
BMI < 18.5 kg/m^2^	(No vs. Yes)	1.27 (0.51–3.17)	0.61		
ADL disability	(No vs. Yes)	0.56 (0.27–1.15)	0.11		
IADL disability	(No vs. Yes)	1.89 (1.13–3.14)	0.01	1.86 (1.13–3.05)	0.01
Smoking habit	(Never vs. Ever)	1.10 (0.53–2.30)	0.79		
Alcohol intake	(Never vs. Ever)	0.59 (0.32–1.08)	0.09		
Regular exercise	(Yes vs. No)	0.94 (0.56–1.56)	0.80		
Handgrip strength	(per 1 kg increase)	1.09 (1.03–1.16)	0.01	1.09 (1.03–1.16)	0.004
Depressive symptoms	(No vs. Yes)	0.94 (0.47–1.87)	0.86		
APOE-ε4 carrier	(No vs. Yes)	0.95 (0.51–1.77)	0.86		

*Abbreviations*: *OR* odds ration, *CI* confidence interval, *MCI* mild cognitive impairment, *NC* normal cognition, *ADL* activities of daily living, *IADL* instrumental activities of daily living, *APOE* apoprotein E

a) The variables were selected by using a backward elimination method, with a retention criterion of* p *< 0.10

Finally, we estimated the 10-year reversion rates for each cognitive status and the mortality rate among the 171 participants with MCI in the 10-year cohort. Figure 2 presents a flowchart illustrating the 10-year changes in cognitive status and mortality among participants with MCI. The 10-year reversion rate from MCI to NC was 18.1% (13.1%–24.6%). Meanwhile, 15.2% (10.6%–21.4%) of participants continued to exhibit MCI, 25.2% (19.2%–32.2%) developed dementia, and 41.5% (34.4%–49.0%) died during the 10-year follow-up period (Fig. 2). We next analyzed the changes in cognitive status at 5-year intervals. Among the 52 participants who reverted from MCI to NC between the 2012–2013 and 2017–2018 surveys, 25 (48.1% [95% CI, 35.0%–61.5%]) maintained NC, 17 (32.7% [21.4%–46.4%]) transitioned back to MCI, and 3 (5.8% [1.9%–16.4%]) progressed to dementia over the subsequent 5 years. We then performed a subgroup analysis of age (< 75 and ≥ 75 years) using the 10-year cohort (Table 4). Among participants aged < 75 years, the 10-year reversion rate from MCI to NC was 37.3% (26.0%–50.2%), while 20.3% (11.9%–32.5%) continued to exhibit MCI, 18.6% (10.6%–30.6%) developed dementia, and 23.7% (14.6%–36.2%) died during the 10-year follow-up period. Among those aged ≥ 75 years, the 10-year reversion rate from MCI to NC was 8.0% (4.2%–14.7%), while 12.5% (7.5%–20.0%) of participants continued to show MCI, 28.6% (21.0%–37.6%) developed dementia, and 50.9% (41.7%–60.0%) died during the 10-year follow-up period (Table 5).

**Table 5:** Subgroup analysis of the 10-year change rates of each cognitive status and mortality by age

Cognitive and health status	No. of events	10-year change rate (95% CI), %
Age < 75 years (*n* = 59)		
Normal cognition	22	37.3 (26.0–50.2)
Mild cognitive impairment	12	20.3 (11.9–32.5)
Dementia	11	18.6 (10.6–30.6)
Death	14	23.7 (14.6–36.2)
Age ≥ 75 years (*n* = 112)		
Normal cognition	9	8.0 (4.2–14.7)
Mild cognitive impairment	14	12.5 (7.5–20.0)
Dementia	32	28.6 (21.0–37.6)
Death	57	50.9 (41.7–60.0)

*Abbreviations*: *CI* confidence interval

## Discussion

The present study demonstrated that the short-term reversion rate from MCI to NC over a 5-year period was 31.3%, based on the 5-year follow-up data pooled from two cohorts that had nearly identical reversion rates. In addition, the study revealed that the long-term reversion rate over the 10-year follow-up period was 18.1%. Intriguingly, among participants who reverted from MCI to NC during the first 5 years, 48.1% maintained NC, 32.7% transitioned back to MCI, and 5.8% progressed to dementia over the subsequent 5 years. Participants aged less than 75 years had higher reversion rates than those aged 75 years or older. Furthermore, younger age, lower systolic blood pressure, absence of diabetes mellitus, preserved IADL, higher handgrip strength, higher MMSE score, higher TBV/ICV levels, and lower WMLV/ICV levels were identified as factors contributing to the reversion from MCI to NC over the 5-year period.

Several population-based prospective studies conducted in Asia and Oceania found that the short-term reversion rates from MCI to NC ranged from 28.0% to 44.0% over a 1- to 6-year follow-up period [5–8]. These rates are almost identical to the 5-year reversion rate observed in the present study. On the other hand, several hospital-based prospective studies showed substantial variability, with the short-term reversion rate from MCI to NC ranging from 8.5% to 22.0% over a 1- or 2-year period [9–11]. These differences in reversion rates to NC between population-based and hospital-based studies may be attributable to variations in study design, including differences in the clustering of risk factors according to the background characteristics of the study populations. In a retrospective cohort study on the long-term reversion rate from MCI to NC, approximately 13% of participants with MCI reverted to NC over a follow-up period ranging from approximately 5 months to almost 15 years (median: 4 years) [12]. This study also found that among participants who reverted from MCI to NC, approximately 19% transitioned back to MCI, 2% developed dementia and 79% retained NC status. The results of this study were almost identical to our present findings using 10-year follow-up data. Collectively, the above previous results and our present findings suggest that approximately 30%–40% of individuals with MCI revert to NC within a short-term period of around 5 years, and among them, about 20%–30% subsequently transition back to MCI. Furthermore, over a long-term period of approximately 10–15 years, around 10%–20% of individuals with MCI may revert to NC.

In this study, several factors—namely, younger age, lower systolic blood pressure, absence of diabetes mellitus, preserved IADL, higher handgrip strength, higher MMSE score, higher TBV/ICV levels, and lower WMLV/ICV levels—were significantly associated with higher odds ratios of reversion from MCI to NC over the 5-year period. Several epidemiological studies, including a meta-analysis, reported that younger age, absence of hypertension and diabetes mellitus, and higher MMSE score were associated with higher likelihood of reversion to NC [5, 7, 9–11, 13–15]. In addition, the PAQUID cohort study reported a clear difference in the reversion rate to NC between MCI participants with IADL impairment and those without (10.7% vs. 34.7%) [16]. A clinical study also found that lower cerebral white matter lesion volume estimated by Fazekas scale was associated with increased reversion to NC [17]. These previous data are also in agreement with our present findings. Other studies have reported significant associations with higher education, the absence of history of stroke and APOE-ε4, and lower GDS score [5, 8–11, 13, 17], but these associations were not found in our study. This may have been due to the small sample size of our study. On the other hand, the present study was the first to show that higher handgrip strength and higher TBV/ICV levels were significantly associated with greater likelihood of reversion from MCI to NC. Notably, other studies have already concluded that these factors are associated with reduced risks of cognitive impairment or dementia [42, 43]. The mechanisms that may drive reversion to NC or progression to dementia remain to be fully elucidated. However, several factors associated with reversion, such as the absence of vascular risk factors and better metabolic profiles, may indicate preserved cerebrovascular integrity and reduced small-vessel disease burden [11, 44]. In addition, cognitive reserve and neuroplasticity might also contribute to recovery of cognitive function among individuals with MCI [45, 46]. Taken together, these findings suggest that the prevention and proper management of lifestyle-related diseases and promotion of healthy lifestyle behaviors may facilitate reversion to NC in older adults with MCI. However, since these studies, like ours, had relatively small sample sizes and short follow-up periods, further investigations with larger scale and longer term follow-up will be needed to verify these findings.

The strengths of this study include the high participation rates in each community-based cross-sectional survey, the consistent methods of assessment of cognitive function, and the high follow-up rate among participants with MCI. However, there are several limitations that should be considered. First, the sample size in this study was relatively small, although this situation was somewhat improved by pooling two cohorts. As a result of the small sample, the accuracy of the estimated change rates in cognitive status and the investigation of contributing factors may have been limited, particularly given the inability to perform multivariable-adjusted analyses or estimate changes in the contributing factors over the 10-year follow-up period. Second, since this study was conducted in a single town in Japan, the generalizability of the findings to other ethnicities or populations may be limited. Third, there may be unmeasured factors associated with reversion to NC that were not accounted for in this analysis. Fourth, the repeated administrations of the MMSE may introduce potential learning effects that could lead to apparent improvement or normalization of scores. However, the diagnosis of MCI in the present study was not based solely on the MMSE. The MMSE was used only as an initial screening tool to detect suspected cases of cognitive impairment, defined as scores below the liberal cutoff of 26 or when subjective or objective cognitive impairment was suspected. Participants who screened positive then underwent further diagnostic evaluation by expert psychiatrists using additional neuropsychological tests. Therefore, the potential influence of learning effects on diagnostic classification is likely limited. Fifth, a potential selection bias may exist in the analysis of factors associated with reversion from MCI to NC. 18 participants excluded from this analysis because follow-up data at 5 years were not available. The baseline characteristics of participants included and excluded from the analysis were compared, showing no significant differences in most demographic, lifestyle, and health-related factors between the two groups, except for the frequencies of lipid-modifying medication use and BMI < 18.5 kg/m^2^, which were higher among participant excluded from the analysis than those included (Table S4). In addition, participants with missing values were excluded from the analysis; especially, 63 participants were excluded due to missing brain MRI data. Participants without brain MRI data were likely to older and had higher mean values of MMSE score, systolic blood pressure and higher frequency of history of cardiovascular disease, as well as ADL and IADL disability than those with brain MRI data (Table S5). Overall, participants excluded from the analysis tended to have higher risk profile for the progression to dementia or death. Finally, some variables regarding lifestyle behaviors were self-reported, which may have led to misclassification or reporting bias. However, these data were collected using standardized questionnaires administered by trained interviewers, which likely minimized this potential bias.

## Conclusions

In a community-dwelling older Japanese population of individuals with MCI, approximately 30% of participants with MCI reverted to NC within a short-term period of 5 years, and about 30% of this group subsequently transitioned back to MCI over the subsequent 5 years. In addition, over a long-term period of 10 years, around 20% of participants with MCI reverted to cognitively normal status. The findings of the present study suggest that the prevention and proper management of lifestyle-related diseases, as well as the promotion of healthy lifestyle behaviors may be associated with reversion to NC in older adults with MCI. However, given the observational nature of this study, the possibility of residual confounding and survivor bias cannot be excluded, and the findings should be interpreted in the context of these methodological considerations. To validate the findings, further large-scale and long-term follow-up studies across multiple countries are needed.

## Supplementary Information

Supplementary Material 1.

Supplementary Material 2.

## Data Availability

The datasets used in the present study are not publicly available, because they contain confidential clinical data on the study participants. However, the data are available on reasonable request and with the permission of the Principal Investigator of the Hisayama Study, Toshiharu Ninomiya.

## Abbreviations

ADL: Activities of daily living
FLAIR: Fluid-attenuated inversion recovery
GDS: Geriatric Depression Scale
IADL: Instrumental activities of daily living
ICV: Intracranial volume
MCI: Mild cognitive impairment
MMSE: Mini-Mental State Examination
MRI: Magnetic resonance imaging
NC: Normal cognition
TBV: Total brain volume
WMLV: White matter lesion volume
