# Supplementary material for: Short-term and long-term reversion rates to normal cognition and their contributing factors among individuals with mild cognitive impairment in a Japanese community: the Hisayama study
Source: BMC Geriatr. 2025 Dec 29;25:1042. doi: 10.1186/s12877-025-06750-7 (PMC12750711; doi:10.1186/s12877-025-06750-7)
Supplement: Supplementary file 1 — Supplementary Material 1. [file 12877_2025_6750_MOESM1_ESM.docx]

**The self**-**administered Health Check-up Questionnaire used in the surveys for this study**

**Q1. Please indicate your highest level of education (years of schooling):**

1. Primary school (6 years or fewer)
2. Junior high school (7–9 years)
3. High school (10–12 years)
4. College/University or higher (13 years or more)

**Q2. How often do you interact (visit, call, etc.) with relatives or friends who are not living with you?**

1. Hardly ever
2. A few times a month
3. A few times a week
4. Almost every day

**Q3. Do you currently (or did you previously) drink alcohol at least once a month?**

1. No
2. Yes
3. Used to drink

9. Unknown

**Q4. Have you ever smoked?**0. Never

1. Yes

9. Unknown

**Q5. In the past month, have you exercised? (Exclude walking while shopping or commuting)** 0. No

1. Yes

**Q6. Have you ever been diagnosed with any of the following? (Please circle all that apply)**

**[Hyperlipidaemia]**
0. No

1. Yes

Are you taking medication for hyperlipidemia?
  0. No
  1. Used to
  2. Occasionally
  3. Regularly

**[Hypertension]**
0. No

1. Yes

Are you taking medication for hypertension?
  0. No
  1. Used to
  2. Occasionally
  3. Regularly

**[Diabetes mellitus]**
0. No

1. Yes

Are you taking medication for diabetes mellitus?
  0. No
  1. Used to
  2. Currently

Do you use insulin or injections?
  0. No
  1. Used to
  2. Currently

**Q7. Please circle the numbers of any illnesses you have had (multiple answers allowed). If none circle 14 at the bottom.**

1-1. Cerebral infarction
  If yes: Date of symptoms (Year / Month / Day) or Unknown
  Hospital visited: (       ) or Unknown
1-2. Cerebral haemorrhage

  If yes: Date of symptoms (Year / Month / Day) or Unknown
  Hospital visited: (       ) or Unknown
1-3. Subarachnoid haemorrhage

  If yes: Date of symptoms (Year / Month / Day) or Unknown
  Hospital visited: (       ) or Unknown
1-4. Other types of stroke

  If yes: Date of symptoms (Year / Month / Day) or Unknown
  Hospital visited: (       ) or Unknown
2-1. Myocardial infarction

  If yes: Date of symptoms (Year / Month / Day) or Unknown
  Hospital visited: (       ) or Unknown
2-2. Angina
3. None of the above
