# Supplementary material for: Short-term and long-term reversion rates to normal cognition and their contributing factors among individuals with mild cognitive impairment in a Japanese community: the Hisayama study
Source: BMC Geriatr. 2025 Dec 29;25:1042. doi: 10.1186/s12877-025-06750-7 (PMC12750711; doi:10.1186/s12877-025-06750-7)
Supplement: Supplementary file 2 — Supplementary Material 2. [file 12877_2025_6750_MOESM2_ESM.docx]

| Table S1. Unadjusted cumulative incidence of MCI-to-NC reversion by presence or absence of each risk factor | | | | | |  |
| --- | --- | --- | --- | --- | --- | --- |
| Variables | Number of reversed cases  /participants | |  | Unadjusted cumulative incidence of reversion (%) | |  |
|  | No | Yes |  | No | Yes |  |
| Age ≥75 years or older | 63/123 | 56/257 |  | 51.2 | 21.8 |  |
| Female sex | 59/181 | 60/199 |  | 32.6 | 30.2 |  |
| Education level (≤ 9 years) | 72/205 | 47/167 |  | 35.1 | 28.1 |  |
| Systolic blood pressure ≥ 140 mmHg | 88/219 | 27/141 |  | 40.2 | 19.2 |  |
| Use of antihypertensive medication | 46/150 | 70/211 |  | 30.7 | 33.2 |  |
| Diabetes mellitus | 91/265 | 25/96 |  | 34.3 | 26.0 |  |
| Serum total cholesterol ≥ 220 mg/dL | 95/269 | 20/91 |  | 35.3 | 22.0 |  |
| Use of lipid-modifying medication | 73/238 | 43/123 |  | 30.7 | 35.0 |  |
| History of cardiovascular disease | 101/314 | 18/66 |  | 32.2 | 27.3 |  |
| BMI <18.5 kg/m^2^ | 110/331 | 6/30 |  | 33.2 | 20.0 |  |
| ADL disability | 110/327 | 9/51 |  | 33.6 | 17.7 |  |
| IADL disability | 69/174 | 50/206 |  | 39.7 | 24.3 |  |
| Ever smoking habit | 65/208 | 50/152 |  | 31.3 | 32.9 |  |
| Ever alcohol intake | 37/142 | 78/218 |  | 26.1 | 35.8 |  |
| Regular exercise | 67/219 | 48/139 |  | 30.6 | 34.5 |  |
| Low handgrip strength < 28 kg (for male), < 18 kg (for female) | 98/250 | 14/90 |  | 39.2 | 15.6 |  |
| Depressive symptoms | 94/274 | 24/93 |  | 34.3 | 25.8 |  |
| APOE-ε4 carrier | 91/301 | 26/68 |  | 30.2 | 38.2 |  |
| Abbreviations: MCI, mild cognitive impairment; NC, normal cognition; BMI, body mass index; ADL, activities of daily living; IADL, instrumental activities of daily living; APOE, apoprotein E; | | | | | | |

| Table S2. Adjusted odds ratios of reversion from MCI-to-NC for each risk factor after excluding deceased cases | | | | |
| --- | --- | --- | --- | --- |
| Variables | Units | Age- and sex-adjusted  OR (95% Cl) of reversion | p-value | FDR  q value |
| MMSE score | (per 1 point increase) | 1.11 (0.98–1.24) | 0.09 | 0.21 |
| ***Demographic, lifestyle, and health-related factors*** | |  |  |  |
| Age | (per 1 year decrease) | 1.11 (1.07–1.15) ^a)^ | <0.001 | <0.001 |
| Sex | (Female vs. Male) | 0.97 (0.60–1.57) ^b)^ | 0.89 | 0.99 |
| Education level (≤ 9 years) | (≥10 years vs. ≤ 9 years) | 1.00 (0.60–1.67) | 0.995 | 0.995 |
| Systolic blood pressure | (per 10 mmHg decrease) | 1.12 (1.00–1.26) | 0.04 | 0.12 |
| Use of antihypertensive medication | (Yes vs. No) | 1.43 (0.86–2.37) | 0.17 | 0.32 |
| Diabetes mellitus | (No vs. Yes) | 1.88 (1.04–3.38) | 0.04 | 0.12 |
| Serum total cholesterol | (per 10 mg/dL decrease) | 1.08 (1.01–1.16) | 0.02 | 0.09 |
| Use of lipid-modifying medication | (Yes vs. No) | 1.20 (0.72–1.99) | 0.49 | 0.68 |
| History of cardiovascular disease | (No vs. Yes) | 1.12 (0.57–2.21) | 0.73 | 0.96 |
| BMI <18.5 kg/m^2^ | (No vs. Yes) | 1.40 (0.57–3.42) | 0.46 | 0.69 |
| ADL disability | (No vs. Yes) | 1.06 (0.49–2.27) | 0.88 | 1.00 |
| IADL disability | (No vs. Yes) | 1.62 (1.02–2.56) | 0.04 | 0.12 |
| Smoking habit | (Never vs. Ever) | 1.00 (0.54–1.83) | 0.99 | 1.00 |
| Alcohol intake | (Never vs. Ever) | 0.63 (0.36–1.10) | 0.10 | 0.22 |
| Regular exercise | (Yes vs. No) | 1.09 (0.67–1.78) | 0.73 | 0.91 |
| Handgrip strength | (per 1 kg increase) | 1.08 (1.02–1.15) | 0.01 | 0.053 |
| Depressive symptoms | (No vs. Yes) | 1.40 (0.77–2.54) | 0.27 | 0.48 |
| APOE-ε4 carrier | (No vs. Yes) | 0.77 (0.42–1.41) | 0.40 | 0.64 |
| ***Brain imaging data*** |  |  |  |  |
| TBV/ICV | (per 1 unit increase) | 1.19 (1.08–1.31) | <0.001 | 0.003 |
| WMLV/ICV | (per 1 unit decrease) | 1.55 (1.06–2.26) | 0.02 | 0.10 |
| Abbreviations: OR, odds ratio; CI, confidence interval; MCI, mild cognitive impairment; NC, normal cognition; MMSE, Mini-Mental State Examination; BMI, body mass index; ADL, activities of daily living; IADL, instrumental activities of daily living; APOE, apoprotein E; TBV, total brain volume; ICV, intracranial volume; WMLV, white matter lesion volume.   1. This value is adjusted for sex. 2. This value is adjusted for age. | | | | |

| Table S3. Adjusted HRs of reversion from MCI-to-NC for each risk factor using the Fine–Gray model | | | | |
| --- | --- | --- | --- | --- |
| Variables | Units | Age- and sex-adjusted  HRs (95% Cl) of reversion | p-value | FDR  q value |
| MMSE score | (per 1 point increase) | 1.14 (1.04–1.24) | 0.01 | 0.03 |
| ***Demographic, lifestyle, and health-related factors*** | |  |  |  |
| Age | (per 1 year decrease) | 1.09 (1.06–1.11) ^a)^ | <0.001 | <0.001 |
| Sex | (Female vs. Male) | 0.91 (0.65–1.27) ^b)^ | 0.57 | 0.63 |
| Education level (≤ 9 years) | (≥10 years vs. ≤ 9 years) | 1.06 (0.75–1.51) | 0.74 | 0.77 |
| Systolic blood pressure | (per 10 mmHg decrease) | 1.10 (1.01–1.19) | 0.03 | 0.08 |
| Use of antihypertensive medication | (Yes vs. No) | 1.38 (0.96–1.96) | 0.08 | 0.21 |
| Diabetes mellitus | (No vs. Yes) | 1.34 (0.89–2.04) | 0.16 | 0.27 |
| Serum total cholesterol | (per 10 mg/dL decrease) | 1.03 (0.99–1.08) | 0.16 | 0.28 |
| Use of lipid-modifying medication | (Yes vs. No) | 1.21 (0.85–1.73) | 0.28 | 0.40 |
| History of cardiovascular disease | (No vs. Yes) | 1.00 (0.61–1.64) | 0.99 | 0.99 |
| BMI <18.5 kg/m^2^ | (No vs. Yes) | 1.54 (0.72–3.31) | 0.27 | 0.40 |
| ADL disability | (No vs. Yes) | 1.59 (0.84–3.00) | 0.16 | 0.30 |
| IADL disability | (No vs. Yes) | 1.60 (1.13–2.27) | 0.01 | 0.04 |
| Smoking habit | (Never vs. Ever) | 1.19 (0.80–1.78) | 0.38 | 0.50 |
| Alcohol intake | (Never vs. Ever) | 0.71 (0.49–1.05) | 0.08 | 0.19 |
| Regular exercise | (Yes vs. No) | 1.16 (0.82–1.65) | 0.40 | 0.50 |
| Handgrip strength | (per 1 kg increase) | 1.07 (1.04–1.11) | <0.001 | <0.001 |
| Depressive symptoms | (No vs. Yes) | 1.40 (0.91–2.15) | 0.13 | 0.27 |
| APOE-ε4 carrier | (No vs. Yes) | 0.86 (0.57–1.30) | 0.47 | 0.55 |
| ***Brain imaging data*** |  |  |  |  |
| TBV/ICV | (per 1 unit increase) | 1.10 (1.04–1.16) | 0.001 | 0.01 |
| WMLV/ICV | (per 1 unit decrease) | 1.41 (1.06–1.86) | 0.02 | 0.06 |
| Abbreviations: HR, hazard ratios; CI, confidence interval; MCI, mild cognitive impairment; MMSE, Mini-Mental State Examination; BMI, body mass index; ADL, activities of daily living; IADL, instrumental activities of daily living; APOE, apoprotein E; TBV, total brain volume; ICV, intracranial volume; WMLV, white matter lesion volume.   1. This value is adjusted for sex. 2. This value is adjusted for age. | | | | |

| Table S4. Baseline characteristics of participants included and excluded from MCI to NC reversion factor analysis | | | |
| --- | --- | --- | --- |
| Variables | Include  (n = 380) | Excluded  (n = 18) | p-value |
| MMSE score, mean (SD) | 24.6 (2.7) | 24.7 (2.5) | 0.85 |
| ***Demographic, lifestyle, and health-related factors*** |  |  |  |
| Age, mean (SD), years | 77.8 (7.0) | 78.7(8.6) | 0.60 |
| Female, % | 52.4 | 55.6 | 0.79 |
| Low education level (≤ 9 years), % | 44.9 | 29.4 | 0.21 |
| Systolic blood pressure, mean (SD), mmHg | 134.9 (21.3) | 135.0 (17.4) | 0.99 |
| Use of antihypertensive medication, % | 58.5 | 46.7 | 0.37 |
| Diabetes mellitus, % | 26.6 | 20.0 | 0.77 |
| Serum total cholesterol, mean (SD), mg/dL | 194.8 (37.3) | 189.1 (30.9) | 0.56 |
| Use of lipid-modifying medication, % | 34.1 | 6.7 | 0.03 |
| History of cardiovascular disease, % | 17.5 | 20.0 | 0.73 |
| BMI, mean (SD), kg/m^2^ | 23.0 (3.6) | 22.1 (4.0) | 0.33 |
| BMI <18.5 kg/m^2^, % | 8.3 | 26.7 | 0.04 |
| ADL disability, % | 13.5 | 27.8 | 0.15 |
| IADL disability, % | 54.2 | 61.1 | 0.57 |
| Ever smoking habit (Current or Former), % | 42.2 | 60.0 | 0.17 |
| Ever alcohol intake (Current or Former), % | 60.6 | 53.3 | 0.58 |
| Regular exercise, % | 38.8 | 33.3 | 0.67 |
| Handgrip strength, mean (SD), kg | 26.3 (8.5) | 25.5 (7.7) | 0.71 |
| Depressive symptoms, % | 25.3 | 17.7 | 0.58 |
| APOE-ε4 carrier, % | 18.4 | 18.2 | 1.00 |
| ***Brain imaging factors*** |  |  |  |
| TBV/ICV, mean (SD), % | 66.5 (3.9) | 66.6 (4.3) | 0.89 |
| WMLV/ICV, geometric mean (geometric SD), % | 0.28 (1.24) | 0.30 (1.64) | 0.77 |

Abbreviations: MCI, mild cognitive impairment; NC, normal cognition; SD, standard deviation; BMI, body mass index; ADL, activities of daily living; IADL, instrumental activities of daily living; MMSE, Mini-Mental State Examination; TBV, total brain volume; ICV, intracranial volume; WMLV, white matter lesion volume.

| Table S5. Baseline characteristics of participants with and without MRI data in MCI to NC reversion analysis | | | |
| --- | --- | --- | --- |
| Variables | Brain MRI data | | p-value |
|  | Presence  (n = 317) | Absence  (n = 63) |  |
| MMSE score, mean (SD) | 24.8 (2.6) | 23.5 (2.8) | 0.001 |
| ***Demographic, lifestyle, and health-related factors*** |  |  |  |
| Age, mean (SD), years | 77.0 (6.7) | 81.6(7.1) | <0.001 |
| Female, % | 52.1 | 54.0 | 0.78 |
| Low education level (≤ 9 years), % | 44.9 | 29.4 | 0.21 |
| Systolic blood pressure, mean (SD), mmHg | 133.4 (20.3) | 144.1 (24.5) | <0.001 |
| Use of antihypertensive medication, % | 56.6 | 69.2 | 0.09 |
| Diabetes mellitus, % | 27.5 | 21.2 | 0.34 |
| Serum total cholesterol, mean (SD), mg/dL | 194.9 (38.4) | 194.5 (30.0) | 0.94 |
| Use of lipid-modifying medication, % | 33.3 | 38.5 | 0.47 |
| History of cardiovascular disease, % | 15.3 | 30.8 | 0.007 |
| BMI, mean (SD), kg/m^2^ | 23.1 (3.6) | 22.6 (3.6) | 0.34 |
| BMI <18.5 kg/m^2^, % | 7.8 | 11.5 | 0.41 |
| ADL disability, % | 9.2 | 35.5 | <0.001 |
| IADL disability, % | 50.2 | 74.6 | <0.001 |
| Ever smoking habit (Current or Former), % | 42.5 | 40.4 | 0.77 |
| Ever alcohol intake (Current or Former), % | 60.7 | 59.6 | 0.88 |
| Regular exercise, % | 39.6 | 34.0 | 0.45 |
| Handgrip strength, mean (SD), kg | 26.3 (8.6) | 26.3 (7.1) | 1.00 |
| Depressive symptoms, % | 24.1 | 33.3 | 0.16 |
| APOE-ε4 carrier, % | 18.4 | 18.6 | 0.96 |
| ***Brain imaging factors*** |  |  |  |
| TBV/ICV, mean (SD), % | 66.5 (3.9) | N/A | N/A |
| WMLV/ICV, geometric mean (geometric SD), % | 0.28 (1.24) | N/A | N/A |

Abbreviations: MCI, mild cognitive impairment; NC, normal cognition; SD, standard deviation; BMI, body mass index; ADL, activities of daily living; IADL, instrumental activities of daily living; MMSE, Mini-Mental State Examination; TBV, total brain volume; ICV, intracranial volume; WMLV, white matter lesion volume.
